# Excited-State Forces with the Gaussian and Augmented Plane Wave Method for the Tamm–Dancoff Approximation of Time-Dependent Density Functional Theory

**DOI:** 10.1021/acs.jctc.4c00614

**Published:** 2024-09-18

**Authors:** Beliz Sertcan Gökmen, Jürg Hutter, Anna-Sophia Hehn

**Affiliations:** † Department of Chemistry, University of Zurich, Winterthurerstrasse 190, 8057 Zurich, Switzerland; ‡ Institute for Physical Chemistry, Christian-Albrechts-University, Max-Eyth-Strasse 1, 24118 Kiel, Germany

## Abstract

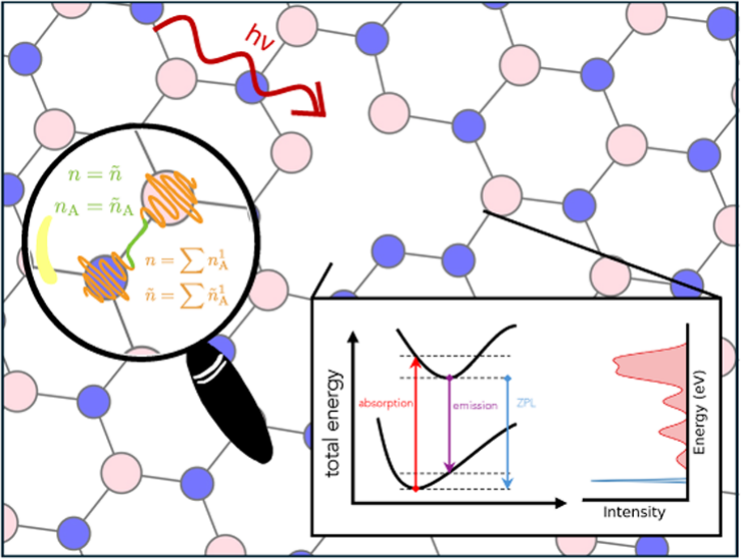

Augmented plane wave methods enable an efficient description of atom-centered or localized features of the electronic density, circumventing high energy cutoffs and thus prohibitive computational costs of pure plane wave formulations. To complement existing implementations for ground-state properties and excitation energies, we present the extension of the Gaussian and augmented plane wave method to excited-state nuclear gradients within the Tamm–Dancoff approximation of time-dependent density functional theory and its implementation in the CP2K program package. Benchmarks for a test set of 35 small molecules demonstrate that maximum errors in the nuclear forces for excited states of singlet and triplet spin multiplicity are smaller than 0.1 eV/Å. The method is furthermore applied to the calculation of the zero-phonon line of defective hexagonal boron nitride. This spectral feature is reproduced with an error of 0.6 eV in comparison to GW–Bethe–Salpeter reference computations and 0.4 eV in comparison to experimental measurements. Accuracy assessments and applications thus demonstrate the potential use of the outlined developments for large-scale applications on excited-state properties of extended systems.

## 1 Introduction

Plane waves (PW) represent the standard basis set choice in solid-state chemistry, implying periodicity by construction and enabling the efficient computation of electrostatic interactions by fast Fourier transformation (FFT) techniques. Linked to intrinsic periodicity is however the major disadvantage of nonlocality, representing a challenge when it comes to describing local fluctuations of the electronic density at the core or when tackling system-specific local features like defects or local charges. Mixed or augmented approaches, that combine plane waves with local basis sets, have therefore been developed extensively, enabling to exploit the advantages of both representations.^1−5^ Mixed schemes expand the global density using both intrinsically periodic plane waves and atom-centered local basis functions, such as Gaussian^1^ or numerical orbitals.^4^ Doing so, the Gaussian and plane wave (GPW) method^1,6,7^ enables to still evaluate Coulomb and exchange correlation (XC) contributions efficiently using FFT, while sparsity of the density matrix can be exploited for contributions of kinetic energy and electron-nuclei interaction. In consequence, the Kohn–Sham (KS) matrix build up in GPW scales as  with system size *N*, which has to be compared with the typical quadratic scaling of PW codes.^8^ However, relying on global densities only, GPW has to replace core electrons with pseudo potentials:^9^ The plane wave representation is sufficiently accurate for feasibly small energy cutoffs when dealing with smooth electron densities in interatomic regions. The description of the heavily oscillating density at the atomic cores would require impracticably high thresholds if not relying on pseudo potentials.^9^ The latter however hinder all-electron computations and thus the modeling of core excitations in e.g., X-ray, nuclear magnetic or electron paramagnetic resonance (NMR or EPR) spectroscopy. Furthermore, in the case of second-row transition metals, it might be necessary to consider localized semicore states in the explicitly treated valence region, requiring high cutoffs for state-of-the-art norm-conserving pseudo potentials.^10^ Augmented plane wave (APW) approaches therefore combine a dual basis-set representation with the standard strategy to divide the system into two regions, interatomic regions, which are still relying on plane waves, and core regions, which are in contrast represented by localized basis sets.^5,11^ Within the extensive research field of APW methods,^2,3,5^ the projector augmented wave (PAW) method by Blöchl^12−14^ introduced the general idea to rely on a linear transformation between pseudo and all-electron wave functions based on projection operators, ensuring that the relevant ground-state or excited-state equations can be reformulated within the auxiliary basis sets, while still expanding integrals over all space. Using projection operators enables to separate the hard local atomic densities from the soft smoothly decaying global density. While the separation of densities can be easily transferred to a separation of local potentials and energies, the treatment of nonlocal Coulomb contributions furthermore requires to introduce screening charges, constructed such that the corresponding multipoles sum to zero, canceling spurious interactions between core and interatomic regions. PAW is closely related to ultrasoft pseudo potentials^15,16^ and is state-of-the-art in leading program packages of solid-state material science.^17−19^

The PAW scheme was also used to extend GPW to the all-electron Gaussian and augmented plane wave (GAPW) approach.^10,20,21^ Starting from an initial implementation for ground-state properties,^20^ GAPW was extended to time-dependent linear response perturbation theory^10,22^ and it was shown that convergence of the plane-wave energy cutoff is accelerated in comparison to GPW, enabling efficiency due to smaller cutoffs while still keeping the GPW scaling for the KS matrix construction. More precisely, a threshold of 200 Ry was shown to be sufficient to ensure converged energies within ≈1μ*E*_h_ and bond lengths fluctuations of less than 20 μÅ. Furthermore, GAPW implementations were generalized for the treatment of core electrons, replacing pseudo potential contributions by corresponding full nuclear potential expressions,^21^ and extended to describe X-ray absorption^23,24^ as well as NMR^25−31^ and EPR spectroscopy.^32−36^ Regarding the computation of hyperfine parameters for the latter spectroscopies, Van Speybroeck and Waroquier *et al.* furthermore suggested a hybrid scheme, which implies an all-electron treatment only for selected nuclei while keeping pseudo potentials for the remaining atoms of the system.^37^

Within this contribution, we present the implementation of GAPW excited-state nuclear gradients for the Tamm–Dancoff approximation (TDA)^38^ of time-dependent density functional theory (TDDFT).^10,39−41^ Our work is based on pioneering work for TDDFT nuclear gradients^22,42−48^ and restricted to TDA, with the latter being often considered as reliable approximation to reproduce peak positions as well as experimental band shapes of emission and absorption spectra.^49^ Relying on a variational Lagrangian formulation of the TDA excited-state eigenvalue problem, the extension to GAPW circumvents the mentioned restrictions of the related GPW implementation^50^ (Section 2.1). Following the fundamental ideas of Blöchl to separate hard and soft densities (Section 2.2) implying screening charges for nonlocal energies (Section 2.3), the required working equations (Section 2.4) involve a discrimination of the GAPW representation for ground- and excited-state densities. Only the former include core compensation charges and thus imply adjusted multipoles for screening. Accuracy of GAPW is assessed for a test set of 35 molecules,^51^ investigating both singlet and triplet vertical excitation energies as well as corresponding excited-state geometries (Section 3.1). Enabling the description of excited-state properties, the GAPW excited-state gradient code is finally applied to compute the zero-phonon line of defective hBN (Section 3.2).

## 2 Methods

### 2.1 Gaussian and Plane Wave (GPW) Framework within Periodic Boundary Conditions

The GPW method relies on both a plane wave and a Gaussian orbital description of the electron density. The total ground-state KS energy within the Γ-point approximation can be written for GPW as
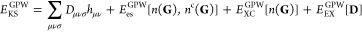
1
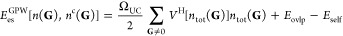
2relying on the density matrix **D** for the description of one-electron and exact exchange contributions, **h** and *E*_EX_, while the plane-wave density *n*(**G**) is used to compute electrostatic and XC contributions, *E*_es_ and *E*_XC_. Ω_UC_ is the unit cell volume. Relying on Ewald summation, a core charge compensation potential has been removed from the external potential contribution to **h** and added to the electronic electrostatic terms. These are divided into Coulomb, self- and overlap energy contributions, thus including core compensation charges *n*^c^(**G**) with the total charge distribution being given as *n*_tot_(**G**) = *n*(**G**) + *n*^c^(**G**), with **G** representing the reciprocal space vectors. More explicit expressions for *E*_ovlp_ and *E*_self_ and their derivatives are summarized in the Supporting Information. The number of plane waves needed for an all-electron description would increase with the square of the nuclear charge and is therefore not feasible in routine applications. In order to avoid this bottleneck, the external potential contributions of **h** are reformulated using pseudo potentials for core electron contributions *E*_PPloc_ and *E*_PPnloc_
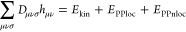
3For an all-electron formulation within GAPW, the pseudo potential operator can be replaced by the corresponding full nuclear potential operator, again subtracting the core charge compensation potential and referring to the conventional Gaussian orbital basis, with explicit formula being given in the Supporting Information. Equations 1 and 2 are indicated with superscript *E*^GPW^ to highlight the GPW approach; for analogous GAPW expressions outlined in the following sections, the corresponding GAPW superscript is omitted for the sake of convenience.

### 2.2 Blöchl’s Projector Augmented Wave method with Gaussian Basis Sets

To circumvent high energy cutoffs for the plane wave basis without being dependent on pseudo potentials, the GAPW method divides the system into core and interatomic regions, *U*_A_ and *U*_I_, with the former being characterized by heavily fluctuating densities requiring high plane wave cutoffs while the latter represent smoothly decaying densities sufficiently described within a small plane wave basis. To describe the core regions within GAPW, the conventional plane wave and Gaussian orbital basis sets of the underlying GPW method are therefore augmented with adjusted basis sets, that are either mimicking a fluctuating “hard” density using a manifold of atomic orbitals generated from primitive Gaussians with large exponents, {φ_μ_(**r**)}, or a smoothly decaying “soft” density with, in contrast, diffuse primitive Gaussians with small exponents, {φ̃_μ_(**r**)}. Furthermore, corresponding hard and soft localized atom-centered atomic orbitals, {χ_μ_(**r**)} and {χ̃_μ_(**r**)}, enable to construct related atomic one-center densities within the core regions, *n̂*_A_^1^(**r**) and *n*_A_^1^(**r**). A description of any arbitrary total density *n*(**r**) in terms of a respective global soft density *ñ*(**r**) is then possible if the smooth atomic densities within the core regions, *ñ*_A_^1^(**r**), are corrected by their hard counterparts, *n*_A_^1^(**r**)
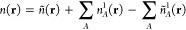
4The superscript indicates that both *n*_A_^1^(**r**) and *ñ*_A_^1^(**r**) represent atomic one-center densities; the subscript labels the atomic cores *A*. The atomic densities are not restricted to the atomic regions, but they cancel within the interatomic domains, *ñ*_*A*_^1^ – *n*_*A*_^1^ = 0 for |**r** – **R**_*A*_| > **R**_at_ ∈ *U*_I_.

For the construction of global soft and hard atomic orbitals and thereon based densities
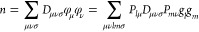
5
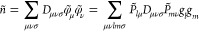
6the fixed contraction coefficients **P** and **P̃** of the primitive Gaussians *g*_*l*_ are chosen to fulfill **P̃** = **P** for small exponents to imply *ñ* = *n* within the interatomic region. Furthermore, the soft contraction coefficients **P̃** of the highest exponent primitives are set to zero according to a given threshold so that ñ is smoothened within the atomic region. The construction of hard and soft atomic densities,
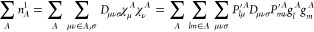
7
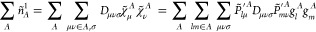
8is borrowing the idea of Blöchl to use projection operators: Within the atomic region, soft and hard coefficients are set equal to their undashed ones, φ̃_μ_ = χ̃_μ_ and *ñ* = *ñ*^1^, and the coefficients **P̃**′ and **P** are constructed relying on projection operators **p**
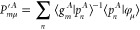
9
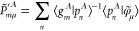
10Indices {μ, ν, κ, λ, ...} denote contracted atomic orbitals φ_μ_, while {*l*, *m*, *n*, *o*...} denote primitive Gaussian functions centered at atom *A*.

### 2.3 Exchange Correlation and Hartree Energies from PAW Densities

Extending the separation into soft and hard contributions for densities to energies is straightforward for local XC contributions, with the GAPW XC energy being given as
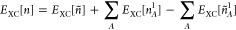
11and corresponding GAPW XC potentials as
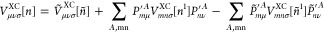
12The first term is calculated on a global grid using FFT techniques and the remaining two contributions are computed numerically using atomic grids. Due to the nonlocal character of the Coulomb operator, the decomposition is however more complex for the electrostatic energy, requiring to introduce atom-dependent screening densities *n*_*A*_^0^
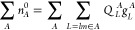
13that generate the same multipole expansion as the local density *n*_*A*_^1^ – *ñ*_*A*_^1^, so that the overall electrostatic interaction cancels to zero, *V*_H_[*n*_*A*_^1^ – *ñ*_*A*_^1^ – *n*^0^] = 0. The multipole expansion is defined as
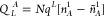
14with *q*^*L*^ representing the multipole moment operator and *N* a normalization constant. The final form of the GAPW Coulomb energy and potential then reads
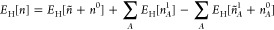
15
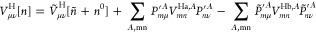
16
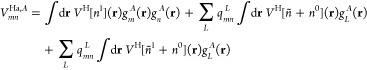
17
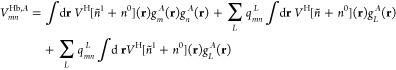
18where *q*_*mn*_^*L*^ is the multipole moment of the local basis product *g*_*m*_(**r**)*g*_*n*_(**r**). Soft contributions can analogously to eq 12 be computed using FFT, while remaining terms are evaluated, in contrast to eq 12, analytically using spherical harmonics. The current formulation is restricted to choosing one screening charge *n*^0^, not discriminating between hard *n*^0^ and soft *ñ*^0^ variants, since the gain in computational cost provided by the latter variant is negligibly small.

When aiming at the description of GAPW excited-state nuclear gradients, both ground-state, excited-state response and difference densities, *n*(**r**), *n*^X^(**r**), and *n*^Z^(**r**), have to be taken into account.

When implying periodic boundary conditions as outlined in section 2.1, the total ground-state density *n*_tot_ thus includes core charges in contrast to *n*^X^ and *n*^Z^, so that the so far discussed equations for the separation of the density, the multipole moments, the GAPW energy and potential have to be adjusted in this case as
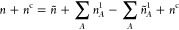
19
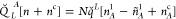
20
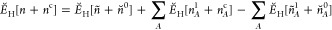
21
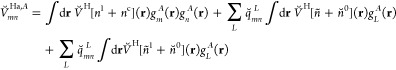
22GAPW intermediates including core charge contributions, such as e.g., the multipole moments **q̆** of eq 20 and thereon relying Coulomb contributions **V̆**^Ha,*A*^ of eq 22, are in the following indexed with the superscript *Ĭ*.

The special form of the GAPW energy functional involves several approximations: The accuracy of the local expansion of the density is controlled by the flexibility of the product basis of primitive Gaussians. As already mentioned, the highest exponents of the soft contraction coefficients are hereby defined by a given threshold. Atomic basis sets are constructed choosing the primitives of the orbital basis, with the option to add further primitives. Core compensation charges are built from one primitive with kind-dependent exponent proportional to the square root of the core charge. The exponent of the screening charge *n*^0^ can be defined on input, otherwise it is computed so that the radius of the primitive Gaussian is smaller than 0.8 a.u. (or 1.2 a.u. in the special case of hydrogen) for a chosen threshold. The definition of the atomic radius **R**_at_ and to what extend the strict conditions on the density are fulfilled in an actual calculation will further determine the accuracy that can be reached.

For convenience, the in the following required derivatives of GAPW Hartree and XC potentials are referring to
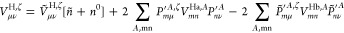
23
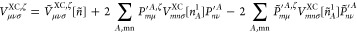
24

### 2.4 Excited-State Nuclear Gradient within GAPW

Excited-state nuclear gradients within the TDA approximation are state-of-the-art,^22,42−48^ standardly formulated based on a variational Lagrangian.^52^ As outlined in the following, it is however crucial to take into account that the definition of the GAPW intermediates differs due to the core charges for ground-state and excited-state contributions as well as contributions stemming from the coupled-perturbed Kohn-Sham (CPKS) equations. For a detailed derivation of TDA ES nuclear gradients, the reader is therefore referred to refs (22) and (44), while we restrict ourselves to shortly summarize the Lagrangian ansatz and to only highlight the adjustments required due to the GAPW framework.

In contrast to the ground-state KS energy *E*_KS_, the excited-state energy Ω_TDA_ within the TDA is not variational with respect to the ground-state MO coefficients **C** and, therefore, the computation of excited-state properties is most conveniently done by setting up a variational Lagrangian *L*,
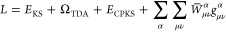
25with the Brillouin condition *E*_CPKS_ ensuring stationarity with respect to the ground-state MO coefficients. Furthermore, when relying on local Gaussian orbitals, various orthogonality conditions **g** have to be ensured. The therefore required additional constraints are introduced via the Lagrange multipliers **W̅** equivalently to GPW formulations^50^ and are given within the Sternheimer formalism as
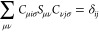
26
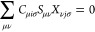
27
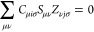
28
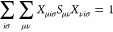
29They refer to the ground-state KS orbitals Φ_*i*σ_(**r**), the solutions to the CPKS equations Φ_*i*σ_^Z^(**r**), and the response orbitals Φ_*i*σ_^X^(**r**) stemming from the TDA eigenvalue problem. Their corresponding basis-set expansion coefficients are denoted *C*_*μiσ*_, *Z*_μ*i*σ_, and *X*_μ*i*σ_, defining response and difference densities *n*(**r**), *n*^X^(**r**), and *n*^Z^(**r**), as well as the corresponding density matrices **D**, **D**^X^ and **D**^Z^,
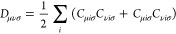
30
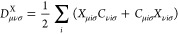
31
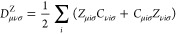
32**S** represents the atomic overlap matrix
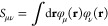
33While eqs 26 and 29 are added with corresponding Lagrange multipliers to the Lagrangian, eqs 27 and 28 are taken into account by adding the projection operator **Q** to the corresponding response equations,
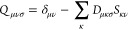
34By enforcing stationarity with respect to the ground-state MO coefficients and by ensuring the mentioned orthogonality conditions, the nuclear gradient is given as partial derivative with respect to the nuclear coordinate ζ, with all orbital expansion coefficients being held fixed at their stationary values
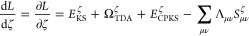
35For the sake of convenience, all contributions to the total ES gradient containing the derivative of the overlap matrix **S**^ζ^ are summarized in terms of matrix Λ, given in more detail in eq 50 of Section 2.4.4. Hard and soft GAPW density representations thereby need to fulfill the conditions
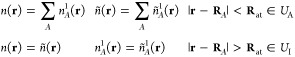


#### 2.4.1 Kohn–Sham Ground-State Contributions Including Core Compensation Charges

More explicitly, the ground-state KS contributions to the GAPW Lagrangian are defined as
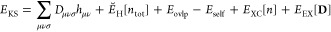
36
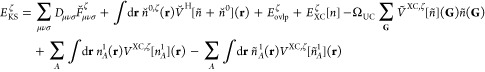
37depending on the GAPW KS matrix **F̆**
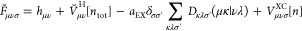
38with the two-electron repulsion integrals being given as
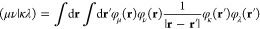
39*a*_EX_ is the global parameter to scale the exact exchange contribution. Computing exact exchange two-electron repulsion integrals in periodic systems is computationally very demanding. The auxiliary density matrix method (ADMM) enables an efficient and rather accurate replacement of these terms. ADMM can be combined with the GAPW method and explicit formulas are provided in the Supporting Information. Furthermore, within GAPW and GPW, the one-electron contributions **h** are computed either referring to eq 3 including kinetic energy and pseudo potential contributions or by replacing pseudo potentials with the all-electron nuclear potential operator, relying for both formulations on the conventional density matrix **D**. As mentioned before, GAPW ground-state electrostatic contributions take into account core compensation charges, thus referring to eqs 19, 20, 21, and 22.

#### 2.4.2 GAPW Expressions for Excited-State Contributions within the Tamm–Dancoff Approximation

Excited-state energies Ω_TDA_ and eigenvectors **X** within the Tamm–Dancoff approximation (TDA) are obtained by solving the eigenvalue problem
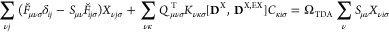
40with the kernel function **K**
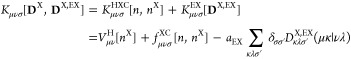
41
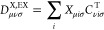
42

In contrast to the ground-state KS matrix contributions, GAPW excited-state electrostatic contributions stemming from the kernel do not take into account core compensation charges, thus referring to eqs 4, 14, 15, and 17. The corresponding derivative furthermore comprises mixed formulations including core charges in the potential while not in the density or vice versa,
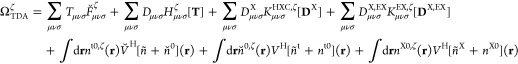
43referring to the unrelaxed difference density matrix **T**
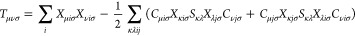
44and the corresponding density *n*^t^(**r**).

#### 2.4.3 Coupled Perturbed Kohn–Sham Equations

In analogy, Coulomb contributions to the CPKS equations have to be adjusted to the GAPW representation by relying on mixed formulations, both for the CPKS linear response equation
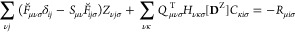
45the corresponding pseudoenergy expression as well as its derivative
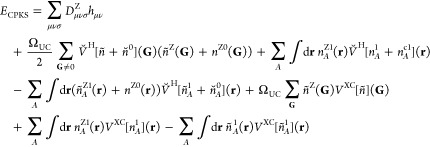
46
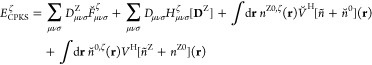
47thus referring to the GAPW KS matrix of eq 38 and the intermediate *H*_μ*νσ*_[**D**^Z^]
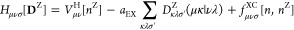
48The right-hand side of the CPKS equations is given as
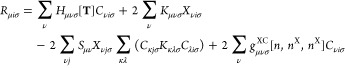
49Even though *n*^Z^ is not including core compensation charges, the KS matrix contributions of eq 45 are based on the total ground-state density *n*_tot_, thus leading to the mixed core compensation gradient terms of eq 47. The right-hand side of the CPKS equations, **R**, is determined by the TDA eigenvalue equations and included GAPW expressions therefore do not include core compensation charges.

#### 2.4.4 Orthogonality Constraints

The additional terms from the orthogonality constraints are relying on the GAPW KS matrix including core charges **F̆**, molecular orbital energies ε_*k*σ_, as well as the GAPW kernels **K** and **H**
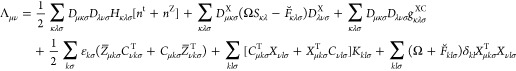
50

## 3 Results and Discussion

### 3.1 Molecular Benchmarks

The GAPW force implementation is assessed with respect to, first, the conventional all-electron molecular quantum chemistry code Gaussian,^53^ and, second, the underlying pseudo potential based GPW implementation in CP2K. Both comparisons elucidate how the GAPW ansatz impacts the overall accuracy of the method, with the first comparison pointing out the error in choosing an additional plane wave basis for the interatomic domains and the second benchmark determining the introduced error by augmenting the basis set with local Gaussians for the atomic regions. For both assessments, we examined ground-state energies and forces, the first three singlet and triplet vertical energies as well as the forces associated with the first singlet and triplet excited states. The former two properties were already benchmarked in refs (10) and (20) and are considered here additionally to enable a comparison of ground-state and excited-state forces and to emphasize consistency. As benchmark systems, we have chosen the molecular test set of Budzák *et al*.,^51^ comprising 35 small molecules containing main-group elements from the first and second rows of the periodic table, as well as sulfur, selenium, chlorine, and bromine. The initial molecular geometries were adopted from our prior work,^50^ wherein we optimized ground-state geometries using the PBE0 functional and the def2-QZVPP basis set^54^ within the Turbomole program package.^55^ For the sake of convenience, our examination of vertical excitation energies is throughout confined to a direct comparison of the first three vertical excitation energies, with states solely identified based on the corresponding electronic structure energy. Furthermore, only nonzero *x*, *y* and *z* components of the ground-state or excited-state forces were considered for statistical analysis. Regarding the overall computational settings, all benchmark computations were performed using the PBE0 density functional. Tight convergence criteria were implied throughout all computations, setting the Schwarz screening threshold for two-electron integrals to 10^–10^ a.u. for both electron repulsion and corresponding derivative integrals. Excited states were converged up to an accuracy of 1.0 × 10^–7^ eV. For the sake of completeness, a comparison to numerical finite differences is furthermore given in the Supporting Information.

#### 3.1.1 Comparison to the All-Electron Gaussian Orbital-Based Code Gaussian

For the first comparison with the all-electron Gaussian reference results, one-electron contributions were computed using all-electron full nuclear potentials in combination with a def2-TZVPP basis set.^56^ The plane wave grid cutoff was set to 400 Ry. Twice as large radial and Lebedev grids were chosen for selenium and bromine and the atomic radius **R**_at_ was adjusted specifically for each element (see Supporting Information). Atomic basis sets were constructed by relying on the orbital basis adding a small number of additional primitive Gaussians.

Maximum errors (Max) and mean absolute errors (MAEs) for ground-state total energies and forces as well as corresponding excited-state analogues are given in Table 1. For ground-state energies, the maximum error is not exceeding 7.10 × 10^–5^ Hartree/atom, and the MAE of 1.75 × 10^–5^ Hartree/atom is negligibly small, emphasizing the agreement of both methods. Deviations for ground-state forces amount up to 0.0293 and 0.0026 eV/Å, respectively, with the most significant deviations for the forces being observed for the C–C triple bonds in acetylene, cyanoacetylene, and diacetylene. The comparison of the first three singlet and triplet excitation energies gives a maximum error for singlet excitations of 0.0102 eV and a MAE of 0.0017 eV. The maximum error and MAE for triplet excitations is of only 0.0015 and 0.0005 eV, respectively. Most importantly, the accuracy of GAPW excited-state forces is comparable to the one of GAPW ground-state forces: The largest errors for the excited-state forces of the first singlet and triplet states are smaller than 0.0303 and 0.0472 eV/Å, respectively, with the most substantial deviations originating again from C–C triple bonds. Corresponding MAEs in forces for the first singlet and triplet states are of 0.0027 and 0.0036 eV/Å, respectively.

**Table 1:** Maximum Errors (Max) and Mean Absolute Errors (MAEs) of Ground-State (GS) Energies (in Hartree/atom) and Forces (in eV/Å) and Vertical Excitation Energies of the First Three Singlet and Triplet Excited States (in eV) and of the Corresponding Forces Associated with the First Singlet or Triplet Excited State (ES) (in eV/Å), Comparing the GAPW Implementation in CP2K with the All-Electron Gaussian Orbital-Based TDA Implementation in Gaussian

	GS		singlet ES		triplet ES	
	energy	force	energy	force	energy	force
Max	7.10 × 10^–5^	0.0293	0.0102	0.0303	0.0015	0.0472
MAE	1.75 × 10^–5^	0.0026	0.0017	0.0027	0.0005	0.0036

#### 3.1.2 GAPW in Comparison to GPW

To compare the GAPW and GPW implementations, the plane wave grid cutoff was adjusted to 800 Ry. Computations were performed relying on Goedecker-Teter-Hutter (GTH) pseudo potentials that were optimized for PBE0 density functional computations and using ccGRB-T orbital basis sets.^9,57^ The augmented basis sets for GAPW were constructed as before by relying on the orbital basis, now of ccGRB-type, adding a small number of primitive Gaussians. A comparative analysis for both ground-state and excited-state computations is provided in Table 2. A correlation plot comparing deviations in the GAPW and GPW excitation energies is given in the Supporting Information. Even though maximum errors are slightly increased and MAEs slightly improved in comparison to the assessment with respect to Gaussian, the overall trend and magnitudes of errors are comparable, emphasizing that sufficient accuracy can be reached with GAPW. Again, errors in ground-state and excited-state forces are of the same magnitude with a maximum error of 0.0932, 0.0931, and 0.0930 eV/Å, respectively.

**Table 2:** Maximum Errors (Max) and Mean Absolute Errors (MAEs) of Ground-State (GS) Energies (in Hartree/atom) and Forces (in eV/Å) and Vertical Excitation Energies of the First Three Singlet and Triplet Excited States (in eV) and of the Corresponding Forces Associated with the First Singlet or Triplet Excited State (ES) (in eV/Å), Comparing GAPW with GPW

	GS		singlet ES		triplet ES	
	energy	force	energy	force	energy	force
Max	3.40 × 10^–5^	0.0932	0.0013	0.0931	0.0008	0.0930
MAE	4.44 × 10^–6^	0.0049	0.0003	0.0045	0.0003	0.0045

### 3.2 Treating Extended Systems: Color Center in Hexagonal Boron Nitride

Hexagonal boron nitride (hBN) is a broadly studied two-dimensional material characterized by its thermal and chemical stability.^58^ Consisting of boron and nitrogen atoms arranged in a hexagonal lattice, hBN belongs to the family of van der Waals materials, sharing similarities with graphene.^59,60^ One of its distinguishing features is its status as a wide band gap semiconductor, with a band gap typically ranging from 5 to 6 eV.^61^ Due to these exceptional properties, hBN has attracted significant attention and found applications in diverse fields such as photonic devices,^62^ fuel cells,^63^ and biomedicine.^64^ Techniques such as chemical vapor deposition, high-temperature annealing of boron nitride precursors, hydrothermal synthesis, and mechanical exfoliation enable the production of hBN in its pure and unaltered form.^60^ On the other hand, the controlled insertion of defects into hBN, such as vacancies or substitutions, can significantly alter its electronic and optical properties. The nitrogen or boron vacancies can introduce electronic states within the band gap of hBN, leading to absorption and emission of light in the visible or near-visible range of the electromagnetic spectrum. The unique capabilities of hBN’s color centers have found application in quantum information processing.^65−69^ The zero-phonon line (ZPL), a distinct feature characterizing electronic transitions without concurrent involvement of vibrational modes or phonons, plays a pivotal role in understanding the luminescent behavior of defects. The ZPL represents a sharp and well-defined emission peak, indicative of a purely electronic transition within the defect. The purity of this electronic transition is particularly significant for applications in quantum optics, where the optically active V_N_N_B_ defect’s ZPL not only provides a clear signature of its luminescent behavior but also serves as a reliable and controllable source of single-photon emission,^65,66,70^ advancing applications in quantum optics and information processing.

**Figure 1:**
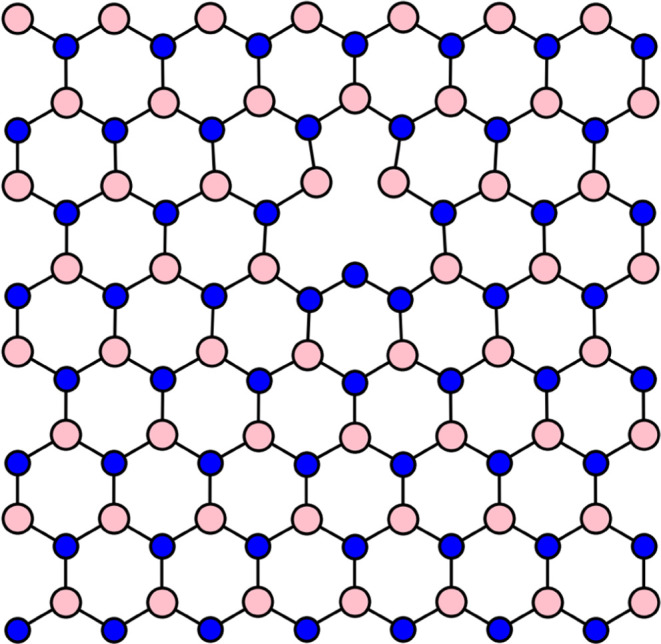
V_N_N_B_ defect in hexagonal boron nitride. There is a vacancy at the nitrogen site and a nitrogen occupying the neighboring boron site. Boron atoms are displayed in pink, nitrogen atoms in blue. The defective nitrogen atom is 0.49 Å above the plane of hBN sheet.

To showcase the applicability range of the GAPW implementation for extended systems, we investigated the V_N_N_B_ defect in hBN, in which a nitrogen occupies a boron site and a neighboring nitrogen site is missing, as visualized in Figure 1. Tran *et al.*(65) showed that the weak interaction between hBN sheets has minimal impact on the properties of the defect, therefore we opted for a single-layer hBN. The monolayer hBN unit cell was replicated to a multiple cell of dimension 7 × 4 × 1 and the monolayer was padded with vacuum in *z*-direction to obtain a simulation box of volume 17.60 × 17.42 × 30 Å^3^. Computations were performed using the HSE06 functional,^71−73^ which is broadly used in studies of semiconductor defects providing band gaps in agreement with experimental observations.^68,74,75^ An open-shell electronic configuration was implied to describe the vacancy appropriately. Shifting from highest-accuracy assessments of molecules to a large-scale application, the computational setup was furthermore adjusted: A MOLOPT basis of triple-ζ quality^76^ was used as primary orbital basis set. Exact exchange contributions are accelerated using ADMM, requiring an additional auxiliary basis of double-ζ quality. The plane-wave grid cutoff was set to 320 Ry.

Upon geometry optimization of the ground-state, the defective nitrogen atom underwent a displacement out of the plane of the hBN sheet by 0.49 Å. Gao *et al.*(77) reported a slightly larger displacement of 0.66 Å obtained from PBE^78^ calculations with pseudo potentials.^79^ As summarized in Table 3, the obtained α- and β-bandgaps of 3.86 and 4.14 eV are in good agreement with PBE-reference values of 3.90 and 4.11 eV.^65^ With 2.93 eV, the first vertical excitation energy deviates by 0.40 eV from the reference,^80^ with the latter however representing Δ*S*CF computations. The zero-phonon line was computed as the difference between the optimized ground-state and first excited-state energy, not including zero-point vibrational energies. The value of 2.56 eV has to be compared with existing experimental values of 2.15 eV^81^ and ∼2 eV,^65^ respectively. Δ*S*CF computations with the HSE06 functional predict ZPL values of 2.05 eV^80^ and 1.90 eV;^82^ PBE results of 1.95 and 1.93 eV^65^ are in close agreement with highly accurate GW-BSE values of 1.92 eV.^77^

**Table 3:** Optical Properties (in eV) Including Band Gaps, Vertical Excitation Energy to the First Excited State (1. ES), and Difference in the First Optimized Ground- and Excited-State Geometry, Zero-Phonon Line (ZPL), of Hexagonal Boron Nitride with the V_N_N_B_ Defect in Comparison to Various Experimental and Computational Studies

		absorption (1. ES)	ZPL	α-band gap	β-band gap
TDA/HSE06	this work	2.93	2.56	3.86	4.14
ΔSCF	Abdi *et al.*(80)	2.53	2.05		
ΔSCF	Li *et al.*(82)		1.90		
TDA/PBE	Tran *et al.*(65)		1.95, 1.93	3.90	4.11
GW-BSE	Gao *et al.*(77)		1.92		
exp.	Grosso *et al.*(81)		2.15		
	Tran *et al.*(65)		∼2		

## 4 Conclusions

The accuracy of our GAPW implementation for excited-state nuclear gradients is compared first, to an all-electron quantum chemistry code and second, to the well-established pseudo potential GPW code with maximum errors not exceeding 3.40 × 10^–5^ Hartree/atom for ground-state energies and 0.09 eV/Å for ground-state forces, 0.01 eV for vertical excitation energies and 0.09 eV/Å for forces associated with first singlet or triplet excited states, for both comparisons. Local features such as the V_N_N_B_ defect in hexagonal boron nitride are accurately reproduced with errors of 0.6 or 0.4 eV in comparison to highly accurate GW-BSE reference computations or experimental data. The presented method developments pave the way for further advancements in the description of localized features and excited core states in solid-state materials, as required e.g., for time-resolved X-ray spectroscopy.

## Supporting Information Available

Periodic boundary conditions and one-electron contributions; XC contributions; exact exchange within the auxiliary density matrix method; atomic radii to define hard and soft densities; comparison of analytical forces to numerical finite differences; correlation plot of GAPW and GPW excitation energies (PDF)
