# Supplementary material for: Excited-State Forces with the Gaussian and Augmented Plane Wave Method for the Tamm–Dancoff Approximation of Time-Dependent Density Functional Theory
Source: J Chem Theory Comput. 2024 Sep 18;20(19):8494–504. doi: 10.1021/acs.jctc.4c00614 (PMC11474744; doi:10.1021/acs.jctc.4c00614)
Supplement: Supplementary file 1 — ct4c00614_si_001.pdf [file ct4c00614_si_001.pdf]

Supplementary information: Excited-State Forces with the Gaussian and Augmented Plane Wave Method for the Tamm-Dancoff Approximation of Time-Dependent Density Functional Theory

Beliz Sertcan Gökmen,[†] Jürg Hutter,[†] and Anna-Sophia Hehn[*,‡]

†*Department of Chemistry, University of Zurich, Winterthurerstrasse 190, 8057 Zurich, Switzerland*

‡*Institute for Physical Chemistry, Christian-Albrechts-University, Max-Eyth-Strasse 1, 24118 Kiel, Germany*

E-mail: hehn@pctc.uni-kiel.de

TABLE OF CONTENTS

1. Periodic boundary conditions and one-electron contributions S3

2. XC contributions S4

3. Exact exchange within the auxiliary density matrix method S5

4. Atomic radii to define hard and soft densities S7

5. Comparison of analytical forces to numerical finite differences S8

6. Correlation plot of GAPW and GPW excitation energies S9

7. References S10

1 Periodic boundary conditions and one-electron contributions

Implying periodic boundary conditions, basis functions, orbitals and potentials have the same periodicity as the system, defined in terms of the general lattice vector $\mathbf{L}$. Electrostatic interactions are described using Ewald summation techniques, introducing core charges as $n^{\rm c}(\mathbf{r}) = \sum_A n^{\rm c}_A(\mathbf{r})$ with

$$n^{\rm c}_A(\mathbf{r}) = -\sum_{\mathbf{L}} Z_A \left(\frac{\alpha^{\rm c}_A}{\pi}\right)^{3/2} e^{-\alpha^{\rm c}_A(\mathbf{r}-\mathbf{R}_A-\mathbf{L})^2} \ , \tag{1}$$

with the atom-dependent Ewald parameter $\alpha^{\rm c}_A$, the effective charge Z_A and position $\mathbf{R}_A$ of atom A. The atomic Ewald compensation potential is then

$$V^{\rm c}_A(\mathbf{r}) = -\sum_{\mathbf{L}} \frac{Z_A}{|\mathbf{r}-\mathbf{R}_A-\mathbf{L}|}\ \mathrm{erf}(\sqrt{\alpha^{\rm c}_A}|\mathbf{r}-\mathbf{R}_A-\mathbf{L}|) \ , \tag{2}$$

and self-energy and overlap contributions to the total electrostatic energy are then given as

$$E_{\rm self} = \sum_A \sqrt{\frac{\alpha^{\rm c}_A}{2\pi}} Z_A^2 \ , \tag{3}$$

$$E_{\rm ovlp} = {\sum_{A,B}}' \sum_{\mathbf{L}} \frac{Z_A Z_B}{|\mathbf{R}_A-\mathbf{R}_B-\mathbf{L}|}\ \mathrm{erfc}\left(\sqrt{\frac{\alpha^{\rm c}_A\alpha^{\rm c}_B}{\alpha^{\rm c}_A+\alpha^{\rm c}_B}}|\mathbf{R}_A-\mathbf{R}_B-\mathbf{L}|\right) \ , \tag{4}$$

where the prime in the first sum imposes $A < B$ for $\mathbf{L} = 0$. The corresponding derivative of the overlap contribution with respect to nuclear coordinate $\zeta = R_{A,i}$ is given as

$$E^{\zeta}_{\rm ovlp} = {\sum_{B}}' \sum_{\mathbf{L}} \left\{ \frac{Z_A Z_B}{|\mathbf{R}_A-\mathbf{R}_B-\mathbf{L}|^3} \mathrm{erfc}\left[\frac{\alpha^{\rm c}_A\alpha^{\rm c}_B}{\alpha^{\rm c}_A+\alpha^{\rm c}_B}|\mathbf{R}_A-\mathbf{R}_B-\mathbf{L}|\right]\right. \tag{5}$$

$$\left. + \frac{2}{\sqrt{\pi}} \frac{\alpha^{\rm c}_A\alpha^{\rm c}_B}{\alpha^{\rm c}_A+\alpha^{\rm c}_B} \frac{Z_A Z_B}{|\mathbf{R}_A-\mathbf{R}_B-\mathbf{L}|^2} \exp\left[-\frac{\alpha^{\rm c}_A\alpha^{\rm c}_B}{\alpha^{\rm c}_A+\alpha^{\rm c}_B}|\mathbf{R}_A-\mathbf{R}_B-\mathbf{L}|^2\right]\right\} \times (R_{A,\zeta}-R_{B,\zeta}-L_\zeta) \ . \tag{6}$$

All-electron formulations for one-electron contributions are given as

$$\begin{aligned}\sum_{\mu\nu\sigma} D_{\mu\nu\sigma} h_{\mu\nu\sigma} &= \sum_{\mathbf{G}} \sum_{i} \frac{1}{2} |c_i(\mathbf{G})|^2 |\mathbf{G}|^2 \\ &+ \sum_{\mu\nu\sigma} \sum_{A} \sum_{\mathbf{L}} D_{\mu\nu\sigma} \int \mathrm{d}\mathbf{r}\, \varphi_\mu(\mathbf{r}+\mathbf{L}) \frac{-Z_A}{|\mathbf{r}-\mathbf{R}_A-\mathbf{L}|} \varphi_\nu(\mathbf{r}+\mathbf{L}) \\ &- \sum_{\mu\nu\sigma} \sum_{A} \sum_{\mathbf{L}} D_{\mu\nu\sigma} \int \mathrm{d}\mathbf{r}\, \varphi_\mu(\mathbf{r}+\mathbf{L}) \frac{-Z_A \mathrm{erf}(\sqrt{\alpha_A^c} |\mathbf{r}-\mathbf{R}_A-\mathbf{L}|)}{|\mathbf{r}-\mathbf{R}_A-\mathbf{L}|} \varphi_\nu(\mathbf{r}+\mathbf{L}) \ .\end{aligned} \tag{7}$$

2 XC contributions

XC potential, kernel and third derivative are defined as

$$V^{\mathrm{xc}}[n](\mathbf{r}) = \frac{\delta E_{\mathrm{xc}}}{\delta n(\mathbf{r})} \ , \tag{8}$$

$$f^{\mathrm{xc}}[n, n'](\mathbf{r}, \mathbf{r}') = \frac{\delta^2 E_{\mathrm{xc}}}{\delta n(\mathbf{r}) \delta n'(\mathbf{r}')} \ , \tag{9}$$

$$g^{\mathrm{xc}}[n, n', n''](\mathbf{r}, \mathbf{r}', \mathbf{r}'') = \frac{\delta^3 E_{\mathrm{xc}}}{\delta n(\mathbf{r}) \delta n'(\mathbf{r}') \delta n''(\mathbf{r}'')} \ , \tag{10}$$

and GAPW formulations and corresponding derivatives for the XC kernel and third derivative are given analogously to the expressions for the XC potential (Eqs. (12) and (27) of the publication),

$$f^{\mathrm{xc}}_{\mu\nu\sigma}[n, n'] = \tilde{f}^{\mathrm{xc}}_{\mu\nu\sigma}[\tilde{n}, \tilde{n}'] + \sum_{A,mn} P'^{A}_{m\mu} f^{\mathrm{xc}}_{mn\sigma}[n^1_A, n'^1_A] P'^{A}_{n\nu} - \sum_{A,mn} \tilde{P}'^{A}_{m\mu} f^{\mathrm{xc}}_{mn\sigma}[\tilde{n}^1_A, \tilde{n}'^1_A] \tilde{P}'^{A}_{n\nu} \ , \tag{11}$$

$$f^{\mathrm{xc},\zeta}_{\mu\nu\sigma}[n, n'] = \tilde{f}^{\mathrm{xc},\zeta}_{\mu\nu\sigma}[\tilde{n}, \tilde{n}'] + 2 \sum_{A,mn} P'^{A,\zeta}_{m\mu} f^{\mathrm{xc}}_{mn\sigma}[n^1_A, n'^1_A] P'^{A}_{n\nu} - 2 \sum_{A,mn} \tilde{P}'^{A,\zeta}_{m\mu} f^{\mathrm{xc}}_{mn\sigma}[\tilde{n}^1_A, \tilde{n}'^1_A] \tilde{P}'^{A}_{n\nu} \ , \tag{12}$$

and

$$g^{\mathrm{XC}}_{\mu\nu\sigma}[n,n',n''] = \tilde{g}^{\mathrm{XC}}_{\mu\nu\sigma}[\tilde{n},\tilde{n}',\tilde{n}''] + \sum_{A,mn} P'^{A}_{m\mu} g^{\mathrm{XC}}_{mn\sigma}[n^1_A, n'^1_A, n''^1_A] P'^{A}_{n\nu}$$

$$- \sum_{A,mn} \tilde{P}'^{A}_{m\mu} g^{\mathrm{XC}}_{mn\sigma}[\tilde{n}^1_A, \tilde{n}'^1_A, \tilde{n}''^1_A] \tilde{P}'^{A}_{n\nu} \ , \tag{13}$$

$$g^{\mathrm{XC},\zeta}_{\mu\nu\sigma}[n,n',n''] = \tilde{g}^{\mathrm{XC}}_{\mu\nu\sigma}[\tilde{n},\tilde{n}',\tilde{n}''] + 2\sum_{A,mn} P'^{A,\zeta}_{m\mu} g^{\mathrm{XC}}_{mn\sigma}[n^1_A, n'^1_A, n''^1_A] P'^{A}_{n\nu}$$

$$- 2\sum_{A,mn} \tilde{P}'^{A,\zeta}_{m\mu} g^{\mathrm{XC}}_{mn\sigma}[\tilde{n}^1_A, \tilde{n}'^1_A, \tilde{n}''^1_A] \tilde{P}'^{A}_{n\nu} \ . \tag{14}$$

3 Exact exchange within the auxiliary density matrix method

Computational costs for the analytical evaluation of the exact exchange integrals are reduced when projecting the exchange contributions onto a much smaller auxiliary basis $\{\check{\mu},\check{\nu},\check{\kappa},\check{\lambda},\dots\}$ as suggested within the Auxiliary Density Matrix Method (ADMM), while alleviating the so-introduced basis-set incompleteness error by adding a GGA density functional correction term $\mathbf{K}^{\mathrm{XGGA}}$,

$$E_{\mathrm{EX}}[\mathbf{D}] = E_{\mathrm{EX}}[\check{\mathbf{D}}] + (E_{\mathrm{EX}}[\mathbf{D}] - E_{\mathrm{EX}}[\check{\mathbf{D}}])$$

$$\approx -a_{\mathrm{EX}}\left[\sum_{\check{\mu}\check{\nu}\sigma} \check{D}_{\check{\mu}\check{\nu}\sigma}\check{D}_{\check{\kappa}\check{\lambda}\sigma}(\check{\mu}\check{\kappa}|\check{\nu}\check{\lambda}) + \sum_{\mu\nu\sigma} D_{\mu\nu\sigma}K^{\mathrm{XGGA}}_{\mu\nu\sigma}[n] - \sum_{\check{\mu}\check{\nu}\sigma}\check{D}_{\check{\mu}\check{\nu}\sigma}\check{K}^{\mathrm{XGGA}}_{\check{\mu}\check{\nu}\sigma}[\check{n}]\right] \ . \tag{15}$$

Auxiliary basis functions $\check{\mu}$ and corresponding density matrices $\check{\mathbf{D}} = \check{\mathbf{U}}\mathbf{D}\check{\mathbf{U}}^{\mathrm{T}}$ and densities $\check{n}$ are obtained using the projection matrix $\check{\mathbf{U}}$, which is constructed from the overlap matrix over the auxiliary basis $\check{\mathbf{S}}$ and the mixed overlap matrix over conventional and auxiliary basis

$\check{\mathbf{V}}$,

$$\check{\mathbf{U}} = \check{\mathbf{S}}^{-1}\check{\mathbf{V}} \ , \tag{16}$$

$$\check{V}_{\check{\mu}\nu} = \int \check{\varphi}_{\check{\mu}}(\mathbf{r})\varphi_{\nu}(\mathbf{r})\mathrm{d}\mathbf{r} \ . \tag{17}$$

For exchange contributions to the ground-state energy and Kohn-Sham matrix, the first-order correction $\mathbf{K}^{\mathrm{XGGA}}$ is given by the GGA exchange potential $\mathbf{V}^{\mathrm{XGGA}}$, while exchange contributions to the excited-state TDA eigenvalue problem and related excited-state contributions to the excited-state nuclear gradient refer to the corresponding GGA exchange kernel $\mathbf{f}^{\mathrm{XGGA}}$,

$$K^{\mathrm{XGGA,\ GS}}_{\mu\nu\sigma}[n] = V^{\mathrm{XGGA}}_{\mu\nu\sigma}[n] \qquad , \qquad K^{\mathrm{XGGA,\ ES}}_{\mu\nu\sigma}[n] = f^{\mathrm{XGGA}}_{\mu\nu\sigma}[n] \ . \tag{18}$$

The GAPW formula of these XC contributions are analogous to the ones given in the previous section of the supplementary information and Eqs. (12) and (27) of the publication. The first analytical term of Eq. (15) is computed within the hard Gaussian orbital basis. As pointed out by Guidon *et al.*,[1] applying the variation principle to Eq. (15) gives a non-trivial expression for the Kohn-Sham matrix involving so-called density purification. The implementation is therefore restricted to their suggestion of a more convenient ansatz, denoted ADMM2, which ignores purification, reprojecting the auxiliary part of the Kohn-Sham matrix $\check{\mathbf{F}}^{\mathrm{ADMM}}$ on the conventional according to

$$\mathbf{F}[\mathbf{D}] \approx \mathbf{F}^{\mathrm{ADMM}}[\mathbf{D}] + \check{\mathbf{U}}^{\mathrm{T}}\check{\mathbf{F}}^{\mathrm{ADMM}}[\check{\mathbf{D}}]\check{\mathbf{U}} \ . \tag{19}$$

Derivatives of auxiliary matrices $\check{\mathbf{M}}$ are obtained by applying the chain rule,

$$\check{\mathbf{M}}^{\zeta} = \check{\mathbf{U}}^{\zeta}\mathbf{M}\check{\mathbf{U}}^{\mathrm{T}} + \check{\mathbf{U}}\mathbf{M}^{\zeta}\check{\mathbf{U}}^{\mathrm{T}} + \check{\mathbf{U}}\mathbf{M}(\check{\mathbf{U}}^{\mathrm{T}})^{\zeta} \ , \tag{20}$$

with the gradient for the ADMM projection matrix being given as $\check{\mathbf{U}}^{\zeta}$,

$$\check{\mathbf{U}}^{\zeta} = \check{\mathbf{S}}^{-1}[\check{\mathbf{V}}^{\zeta} - \check{\mathbf{S}}^{\zeta}\check{\mathbf{U}}] \ . \tag{21}$$

In comparison to Coulomb contributions, exact exchange contributions lack symmetry,

$$\sum_{\mu\nu} X_{\mu k\sigma} C^{\mathrm{T}}_{\nu k\sigma}(\mu\nu|\kappa\lambda) = \sum_{\mu\nu} C_{\mu k\sigma} X^{\mathrm{T}}_{\nu k\sigma}(\mu\nu|\kappa\lambda) \ , \tag{22}$$

$$\sum_{\mu\nu} X_{\mu k\sigma} C^{\mathrm{T}}_{\nu k\sigma}(\kappa\nu|\mu\lambda) \neq \sum_{\mu\nu} C_{\mu k\sigma} X^{\mathrm{T}}_{\nu k\sigma}(\kappa\nu|\mu\lambda) \ , \tag{23}$$

and kernel contributions are therefore described relying on the unsymmetrized density matrix $\mathbf{D}^{\mathrm{X,EX}}$. It should be noted that the current setup within CP2K enables range-separated hybrid functionals with short-range exact exchange such as HSE06 straight-forwardly without further implementation work; however, range-separation involving e.g. long-range Coulomb contributions are not yet included.[2,3]

4 Atomic radii to define hard and soft densities

Table S1: Atomic kinds and corresponding atomic radii $\mathbf{R}_{\mathrm{at}}$ [a.u.] to define atomic and interatomic regions within GAPW.

Kind	$\mathbf{R}_{\mathrm{at}}$	Kind	$\mathbf{R}_{\mathrm{at}}$	Kind	$\mathbf{R}_{\mathrm{at}}$	Kind	$\mathbf{R}_{\mathrm{at}}$	Kind	$\mathbf{R}_{\mathrm{at}}$	Kind	$\mathbf{R}_{\mathrm{at}}$
H	1.00	N	1.40	Al	1.90	K	2.10	Mn	2.10	Ga	2.10
He	1.20	O	1.40	Si	1.90	Ca	2.10	Fe	2.10	Ge	2.00
Li	1.52	F	1.30	P	1.80	Sc	2.10	Co	2.10	As	2.00
Be	1.52	Ne	1.52	S	1.80	Ti	2.10	Ni	2.10	Se	1.90
B	1.52	Na	1.90	Cl	1.60	V	2.10	Cu	2.10	Br	1.90
C	1.52	Mg	1.90	Ar	1.90	Cr	2.10	Zn	2.10	Kr	2.10

5 Comparison of analytical excited-state forces to numerical finite differences

Table S2: Comparison of numerical and analytical excited-state forces for the sulfur atom in Thioformylchloride along x and y directions, with the numerical derivatives being calculated with an increment of 5×10^{-4} a.u. and given in Hartree/a.u., with SCF convergence set to 10^{-8} a.u. and excited-state convergence set to 10^{-9} eV.

GAPW (PBE0/def2-TZVPP)				
Coordinate	$f_{\text{numerical}}$	$f_{\text{analytical}}$	Difference	Error [%]
x	0.05224310	0.05224515	0.00000205	0.00
y	-0.02173634	-0.02173520	0.00000113	0.01
Sum of differences:			0.00000319	0.01
GAPW (PBE0/ccGRB-T/GTH)				
Coordinate	$f_{\text{numerical}}$	$f_{\text{analytical}}$	Difference	Error [%]
x	0.05700512	0.05700619	0.00000107	0.00
y	-0.02399126	-0.02399203	-0.00000077	0.00
Sum of differences:			0.00000183	0.00
GPW (PBE0/ccGRB-T/GTH)				
Coordinate	$f_{\text{numerical}}$	$f_{\text{analytical}}$	Difference	Error [%]
x	0.05702088	0.05702196	0.00000108	0.00
y	-0.02399670	-0.02399747	-0.00000077	0.00
Sum of differences:			0.00000185	0.00

6 Correlation plot of GAPW and GPW excitation energies

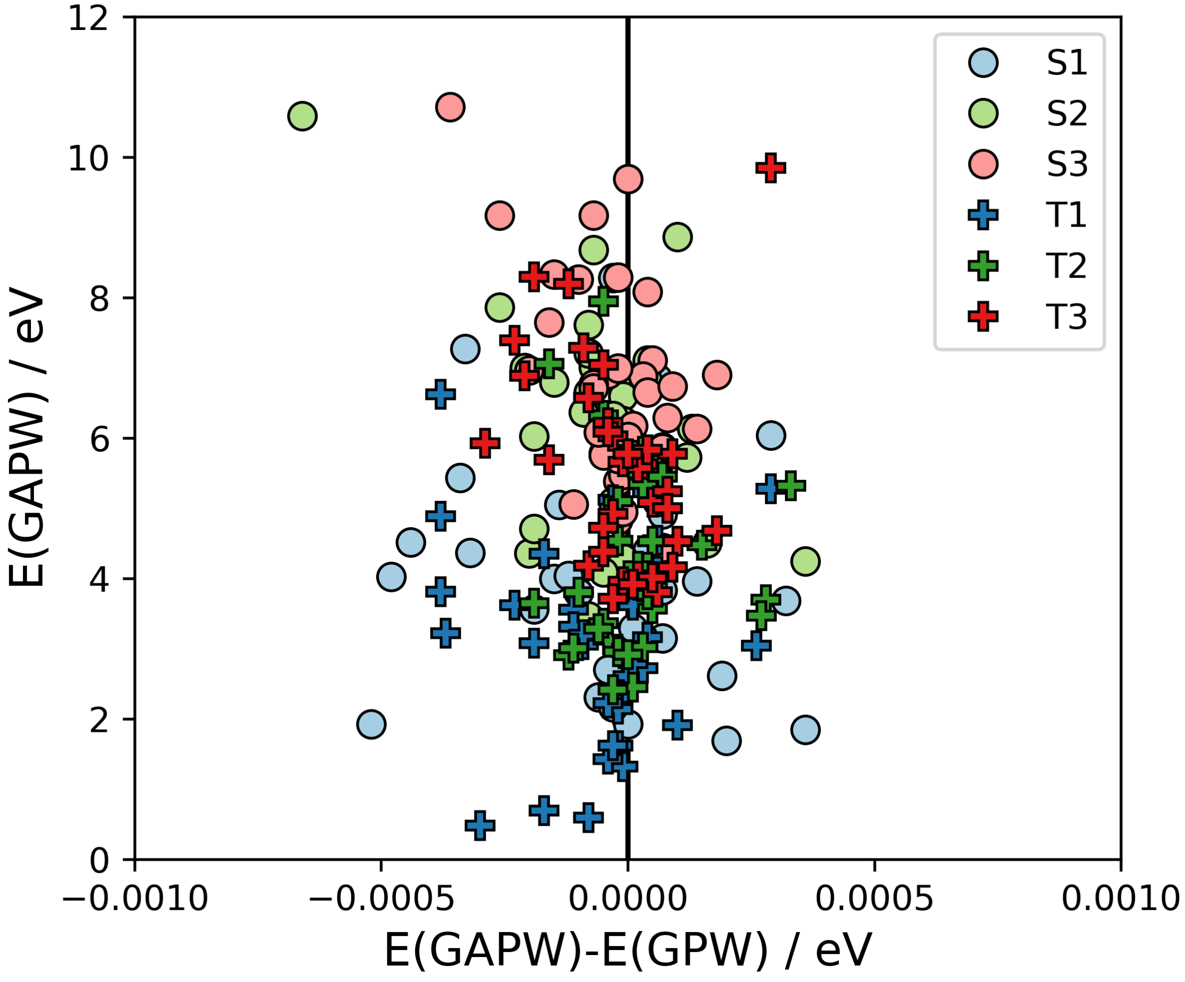

Figure S1: Correlation plot comparing deviations in the first three excitation energies of singlet and triplet multiplicity (denoted S1, S2, S3 and T1, T2, T3, respectively) [in eV] as obtained with GAPW and GPW.

References

(1) Guidon, M.; Hutter, J.; VandeVondele, J. Auxiliary Density Matrix Methods for Hartree-Fock Exchange Calculations. *J. Chem. Theory Comput.* **2010**, *6*, 2348–2364.

(2) Stein, F.; Hutter, J. Double-hybrid density functionals for the condensed phase: Gradients, stress tensor, and auxiliary-density matrix method acceleration. *J. Chem. Phys.* **2022**, *156*, 074107.

(3) Stein, F.; Hutter, J.; Rybkin, V. V. Double-Hybrid DFT Functionals for the Condensed Phase: Gaussian and Plane Waves Implementation and Evaluation. *Molecules* **2020**, *25*, 5174.
